# Complete Genome Sequence of a Novel WU Polyomavirus Isolate from Arkansas, USA, Associated with Acute Respiratory Infection

**DOI:** 10.1128/genomeA.01452-16

**Published:** 2017-01-12

**Authors:** J. L. Kennedy, J. L. Denson, K. S. Schwalm, A. N. Stoner, J. C. Kincaid, T. J. Abramo, T. M. Thompson, E. M. Ulloa, S. W. Burchiel, D. L. Dinwiddie

**Affiliations:** a Department of Pediatrics, Arkansas Children’s Research Institute, University of Arkansas for Medical Sciences, Little Rock, Arkansas, USA; b Clinical and Translational Sciences Center, University of Arkansas for Medical Sciences, Little Rock, Arkansas, USA; c Department of Pharmaceutical Sciences, University of New Mexico College of Pharmacy, Albuquerque, New Mexico, USA; d Department of Pediatrics, University of New Mexico Health Sciences Center, Albuquerque, New Mexico, USA; e Clinical Translational Science Center, University of New Mexico Health Sciences Center, Albuquerque, New Mexico, USA

## Abstract

We report here the complete genome sequence of a WU polyomavirus (WUPyV) isolate, also known as human polyomavirus 4, collected in 2016 from a patient in Arkansas with an acute respiratory infection. Isolate hPyV4/USA/AR001/2016 has a double-stranded DNA genome of 5,229 bp in length.

## GENOME ANNOUNCEMENT

Respiratory tract infections are one of the leading causes for hospitalization in infants and young children. Studies have detailed clinical diseases caused by the most important respiratory tract pathogens, including respiratory syncytial virus, influenza, parainfluenza, rhinovirus, and coronavirus. However, only recently have auxiliary infectious agents of the upper and lower respiratory tract—including human bocavirus (1) and, most recently, WU polyomavirus (WUPyV) (2), also known as human polyomavirus 4 (ICTV species) (3)—gained clinical interest. Most reports of WUPyV have been coinfections or reported in immunosuppressed patients (4–7). However, studies have shown 50% seroconversion WUPyV in the general population by 18 months of age, and others have detected it in children with acute respiratory symptoms (6–10). WUPyV has a double-stranded DNA genome of ~5,200 bp and high sequence identity (4, 11, 12). It is important to document novel nucleotide sequences of all viruses, including WUPyV, to aide researchers and clinicians in the diagnosis and treatment of diseases caused by these potential pathogens.

Here, we describe a novel WUPyV isolate from a nasopharyngeal swab collected from a 13-month-old male (hPyV4/USA/AR001/2016) with no significant past medical history who was seen in the emergency department (ED) of Arkansas Children’s Hospital in Little Rock, Arkansas, USA, on 15 March 2016. His symptoms included nonproductive cough, wheezing, and increased work of breathing. The patient was afebrile, had a heart rate of 160, respiratory rate of 36, and S_A_O_2_ at 95%. He was given vaponephrine and albuterol in the ED. He improved and was able to be discharged home with albuterol as needed.

A nasopharyngeal swab was collected and stored in transport media (Puritan UniTranz-RT; Puritan Diagnostics, USA) after consent was obtained from the parents under a study approved by the University of Arkansas for Medical Sciences Institutional Review Board. An Illumina stranded-RNA sequencing library was created from isolated RNA (Zymo Direct-zol; Zymo Research, USA), and hybridization-based enrichment was performed using the University of New Mexico (UNM) ResVir (respiratory viral) panel probe set designed to be complementary to coding sequence regions of 24 human respiratory viruses. Next-generation sequencing was performed (2 × 75 bp) using V3 sequencing chemistry on an Illumina MiSeq platform. A total of 95,605 reads aligned to the reference sequence NC_009539 and resulted in 99.3% coverage of the reference genome. Generation of a consensus genome sequence of the isolate was performed using CLC Genomics Workbench version 9 and annotated using the VIPR Genome Annotator (13). The 36 missing base pairs of the AR001 genome were completed using PCR and Sanger sequencing.

Alignment of hPyV4/USA/AR001/2016 to NC_009539 revealed a total of 10 variants, of which three were predicted to be nonsynonymous. Phylogenetic analysis of the novel isolate was conducted using neighbor-joining with 1,000 bootstraps to 71 known WUPyV genomes and revealed isolate AR001 grouping closely to isolates W33, NP360, B8649, and B41 with GenBank accession numbers GU296367, GU296402, GU296386, and GU296385. Alignment of our isolate to the closest related sequence, GU296367, showed a total of three variants, of which one was nonsynonymous. The amino acid changing variant was detected in protein ADD50952 (p.Glu89Gln).

### Accession number(s).

The whole-genome sequence of the hPyV4/USA/AR001/2016 isolate has been deposited in GenBank under the accession number KX787894.
